# Factors Affecting Preventive Health Behaviors against COVID-19 in Nursing Students: A Cross-Sectional Study

**DOI:** 10.3390/ijerph19095496

**Published:** 2022-05-01

**Authors:** Young-Mi Jung, Na-Young Kim

**Affiliations:** Department of Nursing, Daegu Haany University, Gyeongsan-si 38610, Korea; youngmi@dhu.ac.kr

**Keywords:** preventive behavior, COVID-19, fear, nursing students

## Abstract

This study was conducted to identify factors affecting preventive health behaviors and to provide basic data for developing coronavirus disease 2019 (COVID-19) prevention and education programs. The participants were 218 students enrolled in two nursing colleges located near Gyeongsang and Jeolla province, Republic of Korea. Data were collected in December 2020 and analyses were conducted using *t*- and Scheffé tests, analysis of variance, Pearson correlation coefficients, and stepwise multiple regression with the SPSS/WIN 25.0 program (IBM Corp., Armonk, NY, USA). The factors affecting preventive health behaviors were fear of infection (β = 0.26, *p* < 0.001), perceived benefits of COVID-19 infection control (β = 0.20, *p =* 0.002), educational needs concerning COVID-19 infection control (β = 0.18, *p* = 0.004), and perceived barriers to COVID-19 infection control (β = 0.16, *p* = 0.011). To improve preventive health behaviors of nursing students against COVID-19, effective and practical education is required, and a systematic infection prevention education program should be developed considering the fear of COVID-19 infection and the perceived benefits and barriers in infection control.

## 1. Introduction

Coronavirus disease 2019 (COVID-19) broke out in Wuhan, Hubei Province, China, in December 2019, and spread rapidly across the world. On 30 January 2020, the World Health Organization declared it a Public Health Emergency of International Concern—the highest alert for infectious diseases. On 11 March 2020, the highest level of stages 1–6 according to the risk level of infectious diseases was declared [1]. The virus has since mutated and continued to devastate the world’s healthcare systems, and, as of 3 February 2022, there were approximately 385 million confirmed cases of COVID-19 and 5,700,000 deaths worldwide [2]. In Korea, the number of confirmed cases exceeds 900,000 and the virus continues to spread [3]. Since February 2020, the infection rate in Korea has risen rapidly with the number of confirmed cases increasing mainly in metropolitan areas, such as the Daegu and Gyeongsangbuk-do regions. The disease is rapidly spreading nationwide again due to the new Omicron variant [4].

According to the Korean Central Disaster and Safety Countermeasure Headquarters, 241 people were infected with COVID-19 among healthcare workers as of 5 April 2020, accounting for 2.4% of all confirmed patients (10,062 people) [5]. Among the confirmed COVID-19-positive medical personnel in Korea at this time, nurses accounted for the highest percentage with 190 positive cases (78.8%) [5]. 

Nurses tend to have the most direct contact with patients among medical personnel at medical facilities and local healthcare institutions that are at the forefront of treating the COVID-19 pandemic. There is thus a higher risk of nurses being exposed to environments where they are vulnerable to infection and of them spreading infectious diseases to others [6,7].

However, nursing students who were training to become nurses were often unable to gain practical clinical experience for the first semester of 2020 due to the COVID-19 pandemic. Compared to nurses who graduated pre-COVID-19, newly graduated nurses may be at higher risk of exposure to infectious diseases due to their lack of proficiency and expertise [8]. Nursing students and recent nursing graduates have been found to display a lack of knowledge and higher levels of stress related to COVID-19 than their predecessors [9]. Therefore, infection control education has become a significant part of the current curriculum for nursing students and together with preventive health behaviors is particularly important during this period of training [7].

To better understand preventive health behaviors, it is necessary to identify the various factors that determine these behaviors. First, knowledge of COVID-19 can affect healthcare workers’ risk perception or prevention strategies [10,11]. This would also be a factor that affects the COVID-19 infection prevention behavior of nursing students [12]. In addition to knowledge of the disease, educational needs are a factor that influences preventive health behavior [13], but the research that has been conducted on nursing students is insufficient. However, a recent study reported a high positive correlation between educational needs and preventive health behaviors or infection control performance [14,15].

Fear of becoming infected can be linked to a person’s anxiety levels, which means that whenever a new infectious disease emerges this can result in the public—and nursing students—experiencing negative emotional effects [16,17,18]. It can, however, also have a positive effect on preventive health behaviors [19,20]. A number of studies have been conducted on mental and emotional difficulties related to COVID-19 [18,21], but the research related to the preventive health behaviors of nursing students is inadequate. Perceived benefits and perceived barriers are also reported as factors related to preventive health behaviors [22,23]. These are used to investigate the influencing factors explaining nurses’ preventive health behaviors, as these are major factors influencing nurses’ infection control [22,23,24]. 

While a number of studies have been conducted related to vaccination in relation to COVID-19 preventive health behaviors [25,26], very few studies have been conducted in which all the variables mentioned above have been investigated with specific reference to the preventive health behaviors of nursing students.

Nurses in the COVID-19 era are required to improve future-oriented education and curricula that can promote global citizenship and cooperative teamwork, and high-level professional nursing services should be provided smoothly [27]. Therefore, this study aimed to provide foundational data for the prevention of COVID-19 transmission amongst nursing students and nurses as well as contribute towards the development of educational programs to decrease transmission by identifying the factors affecting the preventive health behavior of nursing students. More specifically, our goals were as follows.

First, we wished to identify what knowledge of COVID-19 infection control is needed as well as relevant information concerning educational needs related to COVID-19 infection control, fear of COVID-19 infection, benefits and barriers in COVID-19 infection control, and preventive health behaviors of nursing students. Second, we wished to identify differences in relation to knowledge of COVID-19 infection control, educational needs related to COVID-19 infection control, fear of COVID-19 infection, benefits and barriers to COVID-19 infection control, and preventive health behaviors according to the demographic characteristics of nursing students. Third, we wished to investigate the correlation between knowledge of COVID-19 infection control, educational needs related to COVID-19 infection control, fear of COVID-19 infection, benefits and barriers in COVID-19 infection control, and preventive health behaviors of nursing students. Fourth, we wished to identify the factors influencing COVID-19 preventive health behavior in nursing college students.

## 2. Materials and Methods

### 2.1. Study Design

This study adopted a descriptive, questionnaire-based cross-sectional approach to investigate the factors that affect COVID-19 preventive health behavior among nursing students in the Republic of Korea.

### 2.2. Setting and Participants

The participants in the study were nursing college students enrolled in two universities located in the Gyeongsangbuk-do and Jeolla-do provinces of the Republic of Korea. A total of 220 questionnaires were distributed to nursing students and 220 questionnaires were collected. Among them, 218 questionnaires were used in the final data analysis, two questionnaires having been excluded for containing insufficient survey data.

Participants were selected based on their responses. The inclusion criteria comprised participants who: (1) could communicate clearly with other individuals, (2) understood the purpose of the study, and (3) consented to voluntarily and freely take part in the study. The G-Power 3.1.9.7 program (Heinrich-Heine-Universität, Düsseldorf, Germany) was used to calculate the number of participants required for this study. The sample size was estimated at a significance level of 0.05, a statistical power of 0.95, and an effect size of 0.15 for multiple regression analysis, indicating that the minimum number of participants required was 184 [28]. Considering the dropout rate of 15%, the survey was conducted based on the criteria for selecting the study subjects.

### 2.3. Data Collection and Procedure

Before it was distributed to the participants, the survey questionnaire was examined by four nursing college students to determine whether there were any problems with the length, difficulty, and appropriateness of the questions and the response time provided. No issues were raised by the reviewing students at this time, and the responses of these four students to the questionnaire were not included in the survey analysis.

After obtaining approval from the head of the department, a notice was posted on the boards of the respective departments as well as the departments’ websites. The notice advised that the input of research participants was needed for a particular study. The researcher explained the purpose of the study and the contents of the study directly to all nursing students who expressed their intention to participate in the study and then the participants completed the survey questionnaire.

The data for this study were collected during the period December 8 to 31 December 2020, in person and via e-mail. 

### 2.4. Ethical Considerations

This study was approved by the Public Institutional Review Board designated by the Korean Ministry of Health and Welfare to ethically protect study participants (IRB no. P01-202011-21-025). After explaining the purpose and method of the survey to the study participants, written consent forms for participation in the study were distributed to the participants. The researcher explained to the participants that if they did not wish to participate in the study at any stage they could withdraw from the study at any time. It was explained to the participants that their anonymity was guaranteed and that the data collected would be discarded after the completion of the study and not be used for any purpose other than research. Data were anonymized and safely stored in an envelope so as to ensure confidentiality.

### 2.5. Measures

This study used a self-reported questionnaire consisting of six sections: (1) demographic characteristics (age, sex, grade, state of health, satisfaction with school life, major satisfaction, satisfaction with clinical practice, educational experience relating to COVID-19 infection control, intention to participate in COVID-19 infection control education), (2) knowledge of COVID-19 infection control, (3) educational needs concerning COVID-19 infection control, (4) fear of COVID-19 infection, (5) perceived benefits and barriers in COVID-19 infection control, and (6) COVID-19 preventive health behaviors.

#### 2.5.1. Knowledge of COVID-19 Infection Control

Knowledge of COVID-19 infection control was reviewed in relation to the ninth edition of the COVID-19 Response Guideline [29], and two nursing professors developed relevant questions. There were 26 questions in total, 13 of which related to overall COVID-19 knowledge (disease characteristics, prevention of infection path and spread, diagnosis and examination, and treatment regimen), four questions related to the management of the hospital room environment, four questions to the wearing and taking off of personal protective equipment, two questions to the management of medical waste, along with three other questions. After the initial questionnaire was prepared, we assessed the validity of the content by having a group of six infection control nurses, each with more than five years of experience, revise the contents of the questionnaire. They deleted any questions that were ambiguous or duplicated.

The specialized group of reviewing nurses evaluated each question on a four-point Likert scale (4 = “very valid”, 3 = “valid”, 2 = “not valid”, and 1 = “very invalid”). To ensure consistency between the experts, the item-level content validity index (I-CVI) was calculated. The I-CVI is calculated as a response ratio for three and four points per item, and if the I-CVI is 0.78 or higher, it is considered appropriate [30]. The I-CVI values of the 26 questions ranged from 0.17 to 1, and four questions did not exceed the standard value. After the first validity test, three questions with an I-CVI value below the standard or that were considered inappropriate to measure the COVID-19 infection control knowledge of the nursing students were deleted and three questions with overlapping meanings were revised. The content validity of the revised second questionnaire was verified by a team consisting of one professor of infectious medicine, six nurses specializing in infection control, and one nurse. The experts’ I-CVI value met the criteria of 0.88 to 1 for all 16 questions, and the scale-level CVI average (S-CVI/Ave) was 0.94. The final COVID-19 infection control knowledge questionnaire consisted of ten questions in respect of overall COVID-19 knowledge (six questions relating to disease characteristics, two questions in respect of the prevention of infection path and spread, two questions relating to treatment regimen), two questions in respect of the wearing and taking off of personal protective equipment, two questions relating to the management of medical waste, one question relating to the management of the hospital room environment, and one question relating to countermeasures, totaling sixteen questions. The participants had to choose from one of three answers: “yes”, “no”, or “no knowledge”. We allocated one point for the correct answer and 0 points for the incorrect answer or for answering with “no knowledge”. The participants’ total knowledge scores were distributed from a minimum of 0 points to a maximum of 16 points, and the higher the score, the higher the level of the participant’s knowledge. The Kuder–Richardson 20 in this study was 0.98.

#### 2.5.2. Educational Needs Concerning COVID-19 Infection Control

Educational needs concerning COVID-19 infection control were reviewed in relation to the ninth edition of the COVID-19 Response Guideline [29], and two nursing professors developed relevant questions. The questionnaire contained 13 questions, including questions in respect of disease characteristics, diagnosis and examination, treatment regimen, prevention of infection path and spread, wearing and taking off of personal protective gear, quarantine and release from quarantine, the management of the hospital room environment, countermeasures, the reporting of COVID-positive patient statistics and report systems, the management of medical waste, and personal hygiene. As a result of assessing the content validity of this questionnaire, which was reviewed by a group of six nurses specializing in infection control, each with more than five years of experience, the I-CVI value met the criteria of 0.83 to 1 for all 13 questions, and the S-CVI/Ave was 0.92. The score for each question was measured using responses that ranged from 1 (“not at all”) to 5 (“extremely”), and the higher the score, the higher the educational needs for COVID-19 infection control. The Cronbach’s α coefficient of the scale was 0.90.

#### 2.5.3. Fear of COVID-19 Infection

The fear of COVID-19 infection variable was revised and supplemented to suit COVID-19 with an instrument that Lee et al. [31] developed and validated for Middle East respiratory syndrome (MERS) infection in the general public, and two nursing professors developed relevant questions. This questionnaire had its content validity assessed by a group of six infection control nurses, each with more than five years of experience, who reviewed and revised the questionnaire. They deleted questions that were ambiguous or duplicated. The I-CVI values of five of the questions were between 0.67 to 0.83 and one of the questions did not meet the standard. The formulation of this question was revised in accordance with the opinion of experts in the field and it was found to be desirable to clarify and supplement the meaning of the question rather than delete it. The content validity of the revised questionnaire was verified by a group consisting of one professor of infectious medicine, six nurses specializing in infection control, and one nurse. The experts’ I-CVI value met the criteria of 0.88 to 1 for all three questions, and the S-CVI/Ave was 0.92. The score for each question was measured using responses that ranged from 1 (“not at all”) to 5 (“extremely”), and the higher the score, the higher the level of fear of becoming infected with the COVID-19 virus. In the study of Lee et al. [31], the Cronbach’s α coefficient of the scale was 0.77; in this study it was 0.75.

#### 2.5.4. Perceived Benefits and Barriers in COVID-19 Infection Control

The perceived benefits and perceived barriers for the COVID-19 infection control variable were revised and supplemented to suit COVID-19 with the instrument that Lee [32] used to measure perceived benefits and perceived barriers in relation to MERS infection, and two nursing professors developed relevant questions. The questionnaire consisted of three questions in respect of the benefits and three questions relating to the barriers. Its content validity was assessed by a group of six infection control nurses, each with more than five years of experience, who reviewed and revised the questionnaire. They deleted questions that were ambiguous or that had been duplicated. The I-CVI value was 0.67 to 1, and one question did not meet the standard. This question was revised in accordance with the opinion of experts in the field and it was found to be desirable to clarify and supplement the meaning of the question, rather than delete it. The content validity of the revised questionnaire was verified by a group consisting of one professor of infectious medicine, six nurses specializing in infection control, and one nurse. The experts’ I-CVI value met the criteria of 0.88 to 1 for all six questions, and the S-CVI/Ave was 0.94 and 0.92 for perceived benefits and barriers, respectively. The score for each question was measured using responses that ranged from 1 (“not at all”) to 5 (“extremely”), and the higher the score, the higher the level of perceived benefits and barriers in COVID-19. The Cronbach’s α coefficient of the scale of perceived benefits and barriers in COVID-19 infection control in this study were 0.78 and 0.67, respectively.

#### 2.5.5. COVID-19 Preventive Health Behaviors

For COVID-19 preventive health behaviors, two nursing professors prepared the initial questions by reviewing Korean official Basic Guidelines for Distancing in Daily Life [33]. The questionnaire consisted of a total of 11 questions that focused on the five core rules of social distancing in daily life. The content validity of the initial questionnaire was assessed by a group consisting of one professor of infectious medicine, six nurses specializing in infection control, and one nurse. The I-CVI value met the criteria of 0.88 to 1 for all 11 questions, and the S-CVI/Ave was 0.98. The score for each question was measured using responses that ranged from 1 (“not at all”) to 5 (“extremely”), and the higher the score, the higher the level of the COVID-19 preventive health behaviors. In this study, the Cronbach’s α coefficient of the scale was 0.86.

### 2.6. Statistical Analysis

The data collected in this study were processed using the SPSS/WIN 25.0 program (IBM Corp., Armonk, NY, USA), and the data analysis method was as follows. First, descriptive statistics were used for participant demographic characteristics and variables. Second, a *t*-test and analysis of variance (ANOVA) were used to determine the difference in variables related to COVID-19 preventive health behaviors according to demographic characteristics, and the Scheffé test was performed for variables with statistically significant differences. Third, the correlation between variables was analyzed using the Pearson correlation coefficient. Fourth, stepwise multiple regression was used to identify the factors affecting COVID-19 preventive health behaviors.

## 3. Results

### 3.1. Demographic Characteristics

The general characteristics of the nursing students are shown in Table 1. The average age of the participants was 21.17 years and 85.8% of them were females; 31.7% of the participants were in the fourth grade, 22.9% were in the first and third grades, and 22.5% were in the second grade. In respect of their state of health, 74.3% of the participants answered that they were in good health and 20.2% answered that their state of health was moderate. In terms of their satisfaction with university life, 68.2% of the participants answered that their satisfaction levels were good and 27.1% answered that their satisfaction levels were moderate. In respect of their satisfaction with their chosen major, 77.5% of the participants answered that their satisfaction level was good, while 21.1% answered that their satisfaction level was moderate. Concerning their satisfaction level with clinical practice, 60.5% of the participants answered that their satisfaction level was good and 32.8% answered that their satisfaction level was moderate. In response to the question in respect of whether or not they had received COVID-19 infection control education, 71.6% of the participants answered yes. In response to the question of whether they intended to participate in COVID-19 infection control education, 91.3% of the participants answered yes.

### 3.2. Differences in COVID-19 Preventive Health Behaviors by Demographic Characteristics 

The effects of demographic characteristics on COVID-19 preventive health behaviors are shown in Table 1. There were statistically significant differences according to sex (t = −2.759, *p* < 0.01), satisfaction with university life (F = 3.405, *p* < 0.05), and satisfaction with major choice (F = 5.571, *p* < 0.05). The average score of female students was higher than that of male students. As a result of the Scheffé test, in the case of satisfaction with university life and satisfaction with their major choice, the students who responded with “good” had higher average scores than those who responded with “moderate”.

### 3.3. The Mean Scores for COVID-19 Knowledge, Educational Needs, Fear of Infection, Perceived Benefits and Barriers, and Preventive Health Behaviors

As seen in Table 2, the mean score in relation to the nursing students’ knowledge of COVID-19 infection control was 11.57 ± 1.87 out of 16. The mean score in relation to educational needs concerning COVID-19 infection control was 117.50 ± 10.06 out of 130. The mean score in relation to fear of COVID-19 infection was 19.86 ± 3.56 out of 25. The mean score in relation to perceived benefits of COVID-19 infection control was 14.31 ± 1.16 out of 15 and, in relation to perceived barriers to COVID-19 infection control, the mean score was 9.32 ± 2.91 out of 15. The mean score in relation to preventive health behaviors against COVID-19 infection was 47.41 ± 6.12 out of 55.

### 3.4. Correlations among COVID-19 Knowledge, Educational Needs, Fear of Infection, Perceived Benefits and Barriers, and Preventive Health Behaviors

As can be seen in Table 3, the nursing students’ COVID-19 preventive health behaviors and knowledge of COVID-19 infection control (r = 0.227, *p* < 0.01), educational needs concerning COVID-19 infection control (r = 0.220, *p* < 0.01), fear of COVID-19 infection (r = 0.294, *p* < 0.001), perceived benefits of COVID-19 infection control (r = 0.320, *p* < 0.001), and perceived barriers to COVID-19 infection control (r = −0.154, *p* < 0.05) showed a statistically significant correlation.

### 3.5. Factors Affecting COVID-19 Preventive Health Behaviors

Through a stepwise multiple regression analysis, the factors affecting COVID-19 preventive health behaviors among the participants were examined and are presented in Table 4. As a result of identifying basic assumptions and multicollinearity for each step of regression formulation, the Dubin–Watson statistic was 2.044, which closely approached the reference value of 2, so we decided that there was no autocorrelation. The tolerance was 0.911–0.964, which was considerably greater than 0.10. It was confirmed that there was no multicollinearity issue because the variance inflation factor (VIF) ranged from 1.038 to 1.098, which did not exceed the standard value of 10. Therefore, the basic assumptions for performing regression analysis were satisfied. As a result of the analysis, the factors affecting COVID-19 preventive health behaviors were found to be fear of COVID-19 infection (β = 0.26, *p* < 0.001), perceived benefits of COVID-19 infection control (β = 0.20, *p* = 0.002), educational needs concerning COVID-19 infection control (β = 0.18, *p* = 0.004), and perceived barriers to COVID-19 infection control (β = 0.16, *p* = 0.011). The explanatory power of the regression model was 19%, and the regression equation was statistically significant (F = 13.84, *p* < 0.001).

## 4. Discussion

This study was conducted to provide foundational data to assist in the development of COVID-19 education programs by identifying the factors that affect preventive health behaviors in nursing students.

We found statistically significant differences depending on sex, satisfaction with university life, and satisfaction with major choice. 

In a study by Lee [32], who reported on the preventive health behaviors of nursing students related to MERS, statistically significant differences in sex and university life satisfaction were also found, which supports the results of this study. We found that the average score of female students was higher than that of male students, which was consistent with the study of Lee [32] and with another study involving predictors of MERS-related preventive behavior performance among clinical practice students [34]. However, in the study of Kim [35], who investigated preventive behaviors related to MERS in nursing students, there was no significant difference found in infection prevention behaviors according to sex. In a study by Shin [36], who studied hygiene practices related to new infectious diseases among college students, there was no significant difference found according to sex. To increase the effectiveness of an infectious disease education program and to provide customized education, additional research will need to be conducted on preventive health behaviors in relation to infectious diseases among nursing students specifically.

Students who responded that they were satisfied with their university life had higher than average scores than those who responded that they were only moderately satisfied. In terms of whether or not students were aware of the need for COVID-19 infection control education, students who responded with “yes” had higher than average scores than those who responded with “no”, and the same results were found in a similar study by Lee [32]. In the study of Yi et al. [37], the higher the positive attitude toward COVID-19 among nursing and health sciences college students compared to non-health science students, the higher the compliance with preventive behaviors, which also supports the results of this study.

However, in this study, there was a statistically significant difference found according to satisfaction with the student’s choice of major, but in the study of Lee [32], there was no statistically significant difference in major satisfaction. Satisfaction with one’s choice of major is likely to vary from nursing college to nursing college, and other situational factors particular to each nursing student during the COVID-19 pandemic and the MERS epidemic would also affect these results. Further research is needed on the relationship between satisfaction with the choice of major and the preventive health behaviors of nursing students.

We found that the higher the fear of becoming infected with COVID-19, the higher the perceived benefits of COVID-19 infection control, the lower the perceived barriers to COVID-19 infection control, and the higher the educational needs of COVID-19 infection control, the more the level of COVID-19 preventive health behaviors was increased.

A study on the predictors of MERS-related preventive behavior performance among clinical practice students in a tertiary hospital [34] reported similar results to this study. In that study, female students who had received MERS education, who feared becoming infected with MERS, and who had higher knowledge and more positive attitudes about MERS exhibited increased MERS-related preventive behavior performance. In addition, a study that analyzed the factors affecting compliance with preventive behavior performance in relation to COVID-19 as perceived by nursing college students [19] reported that knowledge, anxiety, and experience of self-quarantine were factors influencing compliance with preventive behaviors. In a study by Lee et al. [20], COVID-19 risk perception and anxiety were also found to be factors influencing COVID-19 preventive behaviors. In that study, fear and anxiety appeared as predictors of MERS prevention behavior and influencing factors of COVID-19 prevention behavior, supporting the results of this study. Those with higher levels of fear and anxiety appear to be more vigilant about infection and are more likely to try to prevent it with active behavior. Since fear and anxiety can improve preventive health behaviors, there needs to be a focus on individuals with very low levels of fear of infection. However, increasing fear, which is a negative emotion, and the psychological distress caused by the fear of COVID-19 tends to hinder preventive health behaviors [16]; therefore, preventive health behaviors that do not increase fear of infection with COVID-19 should be developed. Since nursing students are conducting clinical practice during the COVID-19 pandemic, the level of fear of infection will be higher than those who chose different majors, and practical and effective prevention education for nursing students will be needed to reduce these students’ fears.

We found that the higher the perceived benefits and the lower the perceived barriers in relation to COVID-19 infection control, the higher the level of COVID-19 preventive health behaviors. In particular, the influence of perceived benefits was high, and since studies on perceived benefits and barriers among nursing students related to COVID-19 infection are insufficient, we would recommend that further studies on the performance of infection control by nurses be carried out. Perceived benefits appeared to be the most influential factor affecting the performance of infection control in relation to multidrug-resistant organisms among general hospital nurses [22] and in the management of multidrug-resistant organisms among intensive care unit nurses [24], which supports the results of this study. In addition, a study on factors affecting the hand washing practices of clinical nurses [38] showed the perceived benefits and the perceived barriers to be effective, which was in line with the results of this study. A study conducted on clinical nurses [23] reported similar results to those of the present study, showing that infection control awareness and perceived barriers among nurses were factors affecting infection control performance. Various benefits that nursing students will accrue in exercising preventive health behaviors against COVID-19 infection should be emphasized and measures to reduce barriers should be applied when creating educational programs.

The results of this study showed that the higher the educational needs concerning COVID-19 infection control, the higher the level of COVID-19 preventive health behaviors. Although there has been limited research conducted involving nursing students and college students specifically concerning preventive health behaviors, there are some studies that allow for comparison with our study. In a study conducted by Kim et al. [15] on college students, there was a positive correlation found between COVID-19 educational needs and preventive behavior performance. In a study conducted on nurses by Cho et al. [14], the performance of infection control by nurses showed a positive correlation with educational needs related to infection control, which was in line with the results of this study. In a study conducted on nurses working in COVID-19-dedicated hospitals, Kwon [13] reported that educational needs had the greatest influence on COVID-19 infection control performance, which further supports our study findings. During the pandemic, medical workers are required to wear masks and other personal protective equipment [39] and nursing guides for COVID-19 have been developed [40]; however, nurses are still stressed about their workplace responses to the pandemic [41]. It is believed that it will be more difficult for nursing students who are currently being guided by nurses in clinical practice. Nursing students are required to undergo clinical practice despite the COVID-19 pandemic, and those who graduate at this time are exposed to the risk of infection and increased stress. These situations should be considered and a customized education program should be provided based on nursing students’ COVID-19 educational needs.

This study has several strengths. The participants in this study included nursing students who engaged in clinical practice in areas where COVID-19 first had a major effect in South Korea, and it is noteworthy that they participated in this study on preventive health behaviors affecting COVID-19 infection at a time when COVID-19 was so prevalent. Furthermore, along with investigating the influencing factors of preventive health behaviors against COVID-19 infection, which is a topic that has primarily involved nurses thus far, this study focused on nursing students and considered additional factors such as educational needs and perceived benefits and barriers, which are critical influencing factors.

Nevertheless, this study has some limitations. First, this study was conducted within only two provinces of South Korea. The exclusion of nursing students from other provinces may affect the generalizability of the results. Second, a cross-sectional study design cannot establish a causal link between the variables under investigation. Third, regarding the data collection procedure, using two different methods (face-to-face and online) may produce a certain bias in the responses.

## 5. Conclusions

This study analyzed the factors affecting preventive health behaviors in relation to COVID-19 among nursing college students. The key factors affecting COVID-19 preventive health behaviors among nursing students were identified in descending order of importance as follows: fear of COVID-19 infection, perceived benefits of COVID-19 infection control, educational needs concerning COVID-19 infection control, and perceived barriers to COVID-19 infection control. Therefore, to improve the preventive health behaviors of nursing students against COVID-19, effective and practical education is required. Nursing colleges and clinical practice organizations should be systematically organized, taking into consideration nursing students’ fear of COVID-19 infection and the perceived benefits and barriers of infection control when developing COVID-19 infection prevention education programs.

We recommend further research with a larger sample size that could include other variables considered likely to affect COVID-19 preventive health behaviors, as well as further research on the effect of COVID-19 education programs on nursing students with respect to COVID-19 preventive health behaviors.

## Figures and Tables

**Table 1:** Differences in COVID-19 preventive health behaviors by demographic characteristics.

Characteristics	Categories	*N* (%) or M ± SD (Range)	COVID-19 Preventive Health Behaviors (M ± SD)	*t*/F	*p*	Scheffé
Age (yrs)		21.17 ± 1.87 (19–32)				
Sex	Male	31 (14.2)	44.65 ± 5.68	−2.759	0.006	
	Female	187 (85.8)	47.87 ± 6.09			
Grade	1	50 (22.9)	46.50 ± 6.58	0.600	0.616	
	2	49 (22.5)	48.12 ± 5.66			
	3	50 (22.9)	47.60 ± 6.41			
	4	69 (31.7)	47.44 ± 5.93			
State of health	Good	162 (74.3)	47.88 ± 6.02	1.856	0.159	
	Moderate	44 (20.2)	46.18 ± 6.48			
	Poor	12 (5.5)	45.67 ± 5.69			
Satisfaction with school life	Good ^a^	151 (69.2)	48.13 ± 5.91	3.405	0.035	a > b
	Moderate ^b^	59 (27.1)	45.81 ± 6.04			
	Poor ^c^	8 (3.7)	45.75 ± 8.71			
Major satisfaction	Good ^a^	169 (77.5)	48.14 ± 6.04	5.571	0.004	a > b
	Moderate ^b^	46 (21.1)	44.93 ± 5.65			
	Poor ^c^	3 (1.4)	44.33 ± 9.07			
Satisfaction with clinical practice (*n* = 119)	Good	72 (60.5)	47.60 ± 5.73	0.229	0.796	
	Moderate	39 (32.8)	46.97 ± 5.43			
	Poor	8(6.7)	48.37 ± 5.73			
Educational experience relating to COVID-19 infection control	Yes	156 (71.6)	47.40 ± 6.03	−0.034	0.973	
	No	62 (28.4)	47.44 ± 6.39			
Intention to participate in COVID-19 infection control education	Yes	199 (91.3)	47.57 ± 6.10	1.250	0.212	
	No	19 (8.7)	45.74 ± 6.24			

Note: M ± SD = mean ± standard deviation; ^abc^ Scheffé, Scheffé test (post hoc analysis); yrs = years; COVID-19, coronavirus disease 2019.

**Table 2:** The mean scores for COVID-19 knowledge, educational needs, fear of infection, perceived benefits and barriers, and preventive health behaviors.

Variables	M ± SD	Min–Max
Knowledge of COVID-19 infection control	11.57 ± 1.87	6–16
Perceived benefits of COVID-19 infection control	14.31 ± 1.16	11–15
Perceived barriers to COVID-19 infection control	9.32 ± 2.91	3–15
Educational needs concerning COVID-19 infection control	117.50 ± 10.06	83–130
Fear of COVID-19 infection	19.86 ± 3.56	5–25
COVID-19 preventive health behaviors	47.41 ± 6.12	32–55

Note: M ± SD = mean ± standard deviation; Min = minimum value; Max = maximum value; COVID-19, coronavirus disease 2019.

**Table 3:** Correlations among COVID-19 knowledge, educational needs, fear of infection, perceived benefits and barriers, and preventive health behaviors.

Variables	Knowledge ^a^	Educational Needs ^b^	Fear of Infection ^c^	Perceived Benefits ^d^	Perceived Barriers ^e^
COVID-19 preventive health behaviors	0.227 **	0.220 **	0.294 ***	0.320 ***	−0.154 *

Note: *p*-value: * *p* < 0.05, ** *p* < 0.01, *** *p* < 0.001; ^a^: knowledge of COVID-19 infection control, ^b^: educational needs concerning COVID-19 infection control, ^c^: fear of COVID-19 infection, ^d^: perceived benefits of COVID-19 infection control, ^e^: perceived barriers to COVID-19 infection control.

**Table 4:** Factors affecting COVID-19 preventive health behaviors.

Variables	B	S.E.	β	*t*	*p*
(Constant)	13.13	6.11		2.15	0.033
Perceived benefits ^a^	1.07	0.34	0.20	3.12	0.002
Perceived barriers ^b^	−0.34	0.13	−0.16	−2.56	0.011
Educational needs ^c^	0.11	0.04	0.18	2.89	0.004
Fear of infection ^d^	0.45	0.11	0.26	4.13	<0.001
R^2^	0.21				
Adjusted R^2^	0.19				
F(*p*)	13.84 (<0.001)				

Note: B: unstandardized regression coefficient, S.E.: standard errors, β: standardized regression coefficient, R^2^: coefficient of determination, ^a^: perceived benefits of COVID-19 infection control, ^b^: perceived barriers to COVID-19 infection control, ^c^: educational needs concerning COVID-19 infection control, ^d^: fear of COVID-19 infection.

## Data Availability

The data presented in this study are available from the corresponding author upon reasonable request. The data are not publicly available due to privacy or ethical restrictions.
